# Down-regulation of circPVRL3 promotes the proliferation and migration of gastric cancer cells

**DOI:** 10.1038/s41598-018-27837-9

**Published:** 2018-07-04

**Authors:** Han-Dong Sun, Zhi-Peng Xu, Zhi-Qiang Sun, Bin Zhu, Qian Wang, Jian Zhou, Hui Jin, Andi Zhao, Wei-Wei Tang, Xiu-Feng Cao

**Affiliations:** 1 Department of Oncology Surgery, Nanjing First Hospital, Nanjing Medical University, Nanjing, China; 2 Department of Oncology Surgery, Nanjing SIR RUN RUN Hospital, Nanjing Medical University, Nanjing, China; 3 Nanjing Medical University School of Pharmacy, Nanjing, China; 4 Department of Hematology, the First Affiliated Hospital, Nanjing Medical University, Nanjing, China; 5 Department of General Surgery, Nanjing First Hospital, Nanjing Medical University, Nanjing, China

## Abstract

Circular RNA (circRNA) is a key regulator in the development and progression of various types of carcinomas. However, its role in gastric cancer (GC) tumorigenesis is not well understood. The present study aimed to investigate the expression profile and potential modulation of circRNAs on GC carcinogenesis. Human circRNA microarray was performed to screen for abnormally expressed circRNA in GC tissue. Results showed that a decrease in the circPVRL3 expression level was associated with the presence of GC, and also with higher TNM stage and lower overall survival rates compared with that in adjacent noncancerous tissues. *In vitro* assays of the GC cell lines MKN-45 and MGC-803 demonstrated that knockdown of circPVRL3 promoted cell proliferation significantly. Prediction and annotation revealed circPVRL3 was able to sponge to 9 miRNAs and may be also able to have a binding with AGO2, FUS, LIN28A, PTB, and EIF4A3. In addition, based on the structure of internal ribosomal entry sites, open reading frame, and m^6^A modification, circPVRL3 may have the potential ability to encode proteins. Taken together, our study indicated that down-regulation of circPVRL3 could promote the proliferation in gastric carcinoma and have potential to encode protein.

## Introduction

Gastric cancer (GC) is the most common tumor of digestive system and remains the second leading cause of cancer-related deaths worldwide^1^. China’s cancer statistics in 2015 showed that the annual number of GC cases in China was approximately 679,000 and the deaths were approximately 498,000, second only to lung cancer^2^. Despite recent advances in diagnostics and in treatment, there are still a large numbers of GC patients with poor prognosis^3^. Therefore, identifying appropriate molecular biomarkers for early diagnosis and potential targets for GC therapy is in urgent need.

In the last decade, studies have convincingly documented that noncoding RNAs (ncRNAs) participate in regulating of cellular structure, function, and physiological development, and may contribute to the pathogenesis and development of cancer^4^. Of them, circular RNAs (circRNAs), a new class of ncRNAs, were identified in the early 1990s as transcripts with covalently closed loop structures without both 5′-3′ polarities and polyadenylated tails^5^. Recent studies have revealed that there are several noteworthy properties of circRNAs that are produced by backsplicing. Firstly, circRNAs have covalently closed loop structures, which makes them much more stable than liner RNA and insusceptible to degradation by RNA exonuclease or RNase R^6^. Secondly, circRNAs are largely composed of exons, which primarily reside in the cytoplasm and possibly have miRNA response elements (MREs)^7^. Thirdly, circRNAs often exhibit tissue/developmental-stage specific expression^8,9^. Fourthly, circRNAs may bind and sequester RNA-binding proteins (RBPs) or even base-pair with RNAs, resulting in the formation of large RNA-protein complexes (RPCs)^10^. Fifthly, a small portion of engineered circRNAs with internal ribosome entry sites (IRESs), can be translated^11^. Taken together, these properties indicate that circRNAs have the potential to play important roles in transcription and post-transcription and to become ideal biomarkers in the diagnosis of diseases.

Growing evidence has suggested that circRNAs may be involved in the initiation and development of cancers including gastric cancer and could potentially become new biomarkers for cancers^12–14^. For instance, Chen J characterized one circRNA derived from the PVT1 gene and termed it as circPVT1. The expression of circPVT1 was upregulated in GC tissues due to the amplification of its genomic locus and may promote cell proliferation by acting as a sponge for members of the miR-125 family. The level of circPVT1 was observed as an independent prognostic marker for overall survival and disease-free survival of patients with GC^15^. To observe the diagnostic values of circRNAs expression profiles between gastric cancer patients’ plasma and healthy controls, Li T and his colleagues used circRNA microarray and then measured circRNA levels by reverse transcription quantitative polymerase chain reaction (RT-qPCR) and RT-droplet digital PCR (RT-ddPCR), respectively. Results showed a total of 343 differentially expressed circRNAs were found. In cancer and dysplasia tissues, hsa_circ_0001017 and hsa_circ_0061276 were downregulated, significantly associated with distal metastasis. The area under receiver operating characteristic curve in combinative use was 0.966 with 95.5% sensitivity and 95.7% specificity. Moreover, patients with low plasma hsa_circ_0001017 or hsa_circ_0061276 had a much shorter overall survival than those with high levels. Patients whose plasma hsa_circ_0001017 or hsa_circ_0061276 levels recovered to normal after operation had a longer disease-free survival^16^. In addition, another circRNA named hsa_circ_002059, was found to be significantly down regulated in gastric cancer plasma and correlated with distal metastasis, tumor-node-metastasis (TNM) stage, gender and age, which might be a potential novel and stable biomarker for the diagnosis of gastric carcinoma with a better sensitivity and specificity^17^.

The present study utilized human circRNA microarray analysis to screen circRNA expression profiles in GC tissues, and discovered a significantly down-regulated circRNA hsa_circ_0066779 (GSE100170 in the GEO database). Has_circ_0066779 is in gene symbol *PVRL3* and thus, we named it as circPVRL3. Based on the microarray findings, a series of functional validation experiments were performed to explore the role of circPVRL3 in GC.

## Materials and Methods

### Ethics statement

Written informed consent was obtained from each patient before recruitment, and the medical ethics committee of Nanjing Medical University approved the study protocol. All methods were performed in accordance with the relevant guidelines and regulations.

### Sanger sequencing

The amplifcation products were inserted into a T-vector for Sanger sequencing to determine their full-length. The divergent primers were designed to confirm the back-splice junction of circPVRL3: 5′-AAGACCTATTTCAG-3′ (sense) and 5′- ATGCTCCTGAAGTA-3′ (antisense). The primers were synthesized by Invitrogen (Shanghai, China), and Sanger sequencing was performed by Realgene (Nanjing, China).

### RNase R treatment

A total of 2 mg RNA was incubated for 20 min at 37 °C with or without 3 U mg^−1^ of RNase R. RNeasy MinElute cleaning Kit (Qiagen) was used to purify the resulting RNA.

### Patients and clinical tissue samples

A total of 62 GC tissues and corresponding non-tumor tissues were obtained from GC patients who was hospitalized in Department of Oncology Surgery, Nanjing Medical University Nanjing Hospital, Nanjing, China during the period of time (January 2007 through July 2017). All of the patients were naive-radiotherapy or -chemotherapy before enrollment, and their tissue specimens were immediately stored in RNA-fixer Reagent after removal from patients’ stomachs and were kept at −80 °C in a refrigerator until analysis. Following the principle, the paired adjacent non-tumor tissues were confirmed to have no tumor cells through pathological analysis, and were localized at 5 cm away from the edge of the GC site. According to the tumor-node-metastasis (TNM) staging system of the International Union Against Cancer (v.8; 2016), all tumors were staged accurately.

### Cell line, cell culture, and transfection

Human GC cell lines MKN-45, MGC-803, SGC-7901, and AGS were established from samples extracted from human GC patients by Shanghai Institutes for Biological Sciences, Chinese Academy of Sciences, Shanghai, China. The human gastric epithelial cell line GES-1 was obtained from the Cancer Institute and Hospital of the Chinese Academy of Medical Sciences (Beijing, China). MKN-45 and MGC-803 cells were transfected with 50 nM si-circPVRL3 or si-NC using Lipofectamine 2000 (Invitrogen, Carlsbad, CA, USA) according to the manufacturer’s kit instructions. The target sequence for circPVRL3 siRNAs was as follows: siRNA-1: 5′-TCAGATGCTCCTGAAGTTT-3′; siRNA-2: 5′-ACCTATTTCAGATGCTCCT-3′; siRNA-3: 5′-TTCAGATGCTCCTGAAGTT-3′. After 48 h, knockdown of circPVRL3 was confirmed via quantitative real-time PCR (qRT-PCR).

### RNA Isolation, Reverse Transcription, and qRT-PCR

Total RNA was extracted from clinical specimens or cell lines using the Trizol reagent (Invitrogen). RNA was reverse transcribed into cDNA with the Prime-Script TM one-step RT-PCR kit (Generay, Shanghai, China). CircPVRL3 expression level was determined by qRT-PCR using the following primer pair: Forward (F): 5′-CCCACACATAAACCACCTCCTC-3′and Reverse (R): 5′-TGCATCAGCATTACATTTGAGA-3′. Glyceraldehyde 3-phosphate dehydrogenase (GAPDH) was used as a control, with a primer pair: 5′-GCATCCTGGGCTACACTG-3′ (F) and 5′-ACTTCAGGAGCATCTGAAATAGGT-3′ (R). The expression level of PVRL3 mRNA was determined by qRT-PCR using a primer pair 5′-GCAGTTCACCATCCCCAATATG-3′ (F) and 5′-TCCAAGCGGGAATGTAACAGC-3′ (R). ABI7500 System was applied into all qRT-PCR reactions. To accurately verify fold changes of mRNA expression of circPVRL3 tested in GC tissues, calculated Ct values were normalized against those of GAPDH that was amplified from the same sample (ΔCt = Ct_tested_ − Ct_GAPDH_), and the −ΔCt method was used to estimate the relative expression value. Each sample was run in triplicates, and all reactions were repeated three times independently to ensure the reproducibility of all the data.

### CCK-8 assay

The CCK8 assay was applied to measure relative cell growth rate. MKN-45 and MGC-803 cells were plated at 3 × 10^3^ cells per well in 96-well plates with four wells for each condition, transfected with si-NC or si-circPVRL3. The absorbance at 450 nm was measured to evaluate the cell viability every 24 h using a microplate reader (MD, M2e,USA).

### Immunofluorescence staining

A coverslip was added on a 12-well plate and the cells were cultured in culture media to about 50% confluence. Then media was aspirated from plates with phosphate-buffered saline (PBS) washed twice. Cells were fixed with 4% paraformaldehyde (PFA) for 30 min at room temperature. Washed by phosphate buffered saline 3 times, cells were treated with PBS-0.2% Triton-X100 for 10 minutes. After blockade for 1 h with PBS-10% donkey serum, cells were stained with primary antibody (1:1000,rabbit anti-Ki-67,Invitrogen,180191Z) diluted with PBS-10% donkey serum and 0.2% Triton-X100 by forming a drop on the coverslip at 4 °C overnight. On the second day, after washed for 10 min 3 times with PBS, cells were stained with conjugated secondary antibody (1:1000,rabbit IgG,Life Technologies,A21206) diluted with 10% donkey serum for 1 h at room temperature. Coverslip was mounted with Fluoromount-G (SouthernBiotech, 0100-01) for fluorescent imaging. Images were acquired using an Eclipse 80i Fluorescence Microscope.

### Nucleus-cytoplasm fractionation

Firstly, 1 × 10^6^ MKN-45 cells were washed twice with PBS. The cell layer was scraped into 500 μl of PBS and centrifuged 5 min at 500 × g at 4 °C. The supernatant was cleared, and this wash step was performed twice. Both nuclear and cytoplasmic RNA from cultured MKN-45 cells were isolated by PARIS KIT 50 RXNS (life, AM1921) followed manufactuere’s instruction. GAPDH processed mRNA was detected in isolated RNAs as control for nuclear RNA and cytoplasm RNA, respectively. Biological triplicates were carried out and followed by qRT–PCR to detect abundance of circPVRL3 and PVRL3 mRNA. 45S and 7SL as marks have to be used to show an efficient nuclear/cytoplasmic RNA separation^18^.

### Scratch wound assay

MKN-45 and MGC-803 cells were transfected with 50 nMsi-circPVRL3 or si-NC. At 24 hours after transfection, wounds were created using a 1 ml pipette tip when cell confluence reached approximately 80%. The cells were then rinsed to remove floating cells and debris. Wound healing was observed at different time points and photographed at the same time.

### Annotation of some potential functions of circPVRL3

The IRES was annotated according to pioneer IRES search system (IRSS), with an accuracy rate of 98.53%, 90.80%, 82.36% and 80.41% for IRES group 1, 2, 3, and 4, respectively. The ORF was predicted according to circular RNA interactome website. N6 -methyladenosine (m^6^A) modification preferably occurs in the consensus motif “RRm6 ACH” (R = G or A; H = A, C or U). Therefore,we matched the possible sequences to the circPVRL3 including GGACA,GAACA,GGACC,GGACT,GAACT,AAACA,AAACC,AAACT,AGACA,AGACC,AGACT, and GAACC.

### Statistical analysis

An independent *t*-test was used to analyze the comparison of continuous data and the chi-square test was applied into categorical data. A receiver operating characteristic (ROC) curve was performed to evaluate its diagnostic value. Kaplan–Meier survival analysis and log-rank tests were performed to evaluate the correlation between circPVRL3 expression and prognosis of patients with GC. All statistical analysis was performed using SPSS17.0 (Chicago, USA). For all results, P < 0.05 was considered statistically significant.

### Availability of data and material

All the data and material have been agreed by authors and Nanjing first hospital.

## Results

### circRNA expression profiles in GC tissues relative to adjacent nontumorous tissues

**Figure 1:**
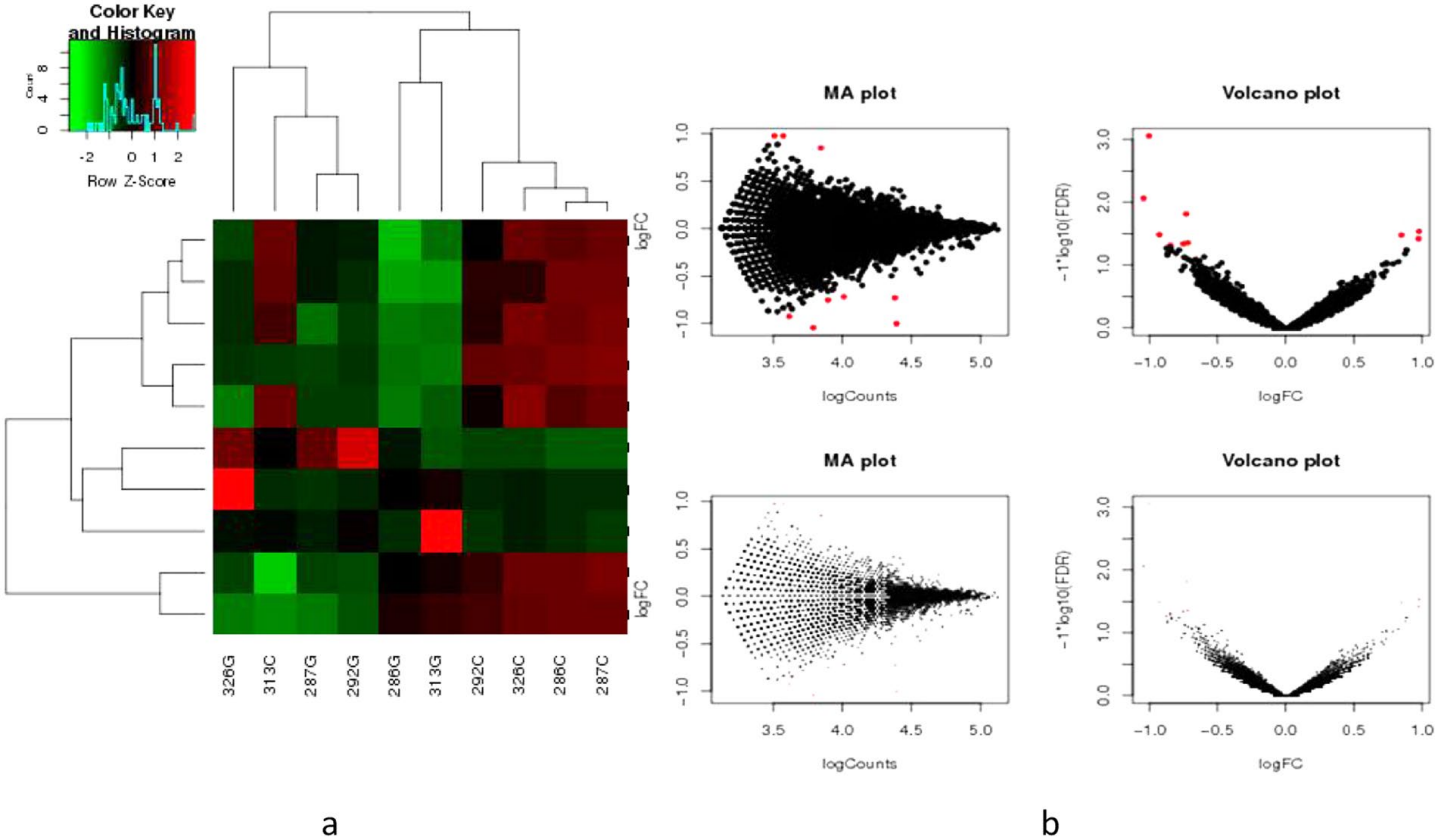
Profiling of circular RNAs in gastric normal and cancerous tissues. (**a**) Heat map showed the selected 5 up-regulated and 5 down-regulated circRNAs. (G for GC, and C for control adjacent nontumorous tissues). Each column represents the expression profile of a tissue sample, and each row corresponds to a circRNA. “Red” indicates higher expression level, and “green” indicates lower expression level. (**b**) Scatter plot showed the visualizing circRNAs different expression in GC tissues and adjacent noncancerous tissues.

A total of 5 GC tissues and their matched non-GC tissues were collected and screened for dysregulated circRNA using human circRNA microarray. A total of 713 circRNAs were differentially expressed in GC tissues vs. non-GC tissues among all the candidate circRNAs detected in both GC and non-GC tissues according to the *t*-test. Of 713 circRNAs, 191 were significantly up-regulated in GC tissues, whereas 522 were significantly down-regulated. We selected a total of 10 upregulated and downregulated circRNAs from high rank to low based on the multiple fold difference between the expression of cancer and adjacent noncancerous tissues and made out a heat map as well as scatter plot as shown in Fig. 1a,b. Subsequently, we used qRT-PCR to detect the expression levels of these dysregulated circRNAs in gastric cancer and noncancerous tissues from our hospital and found that compared to other 9 circRNAs, the expression of circPVRL3 was significantly decreased and had significant correlation with clinicopathological data. In addition, bioinformatics predictions revealed that circPVRL3 might have a rich biological function and was selected as a candidate target for GC for further study.

### The Biological structure of circPVRL3

**Figure 2:**
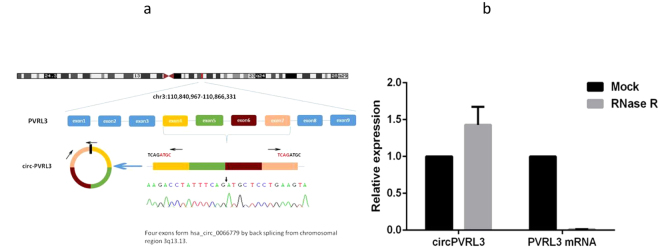
The biological structure of circPVRL3. (**a**) Schematics showes that circPVRL3 is derived from PVRL3 exons 4–7 (**b**) qRT–PCR for the abundance of circPVRL3 and *PVRL3* mRNA in GC cells treated with RNase R. The amount of circPVRL3 and *PVRL3* mRNA were normalized to the value measured in the mock treatment.

We next investigated the mechanisms by which circPVRL3 was formed. Figure 2a indicated that how circPVRL3 is derived from the exons 4, 5, 6, and 7 of the gene *PVRL3*. Resistance to digestion with RNase R exonuclease further demonstrated that circPVRL3 is a stable circular RNA (Fig. 2b).

### Expression of circPVRL3 and clinicopathologic information in patients with GC

**Figure 3:**
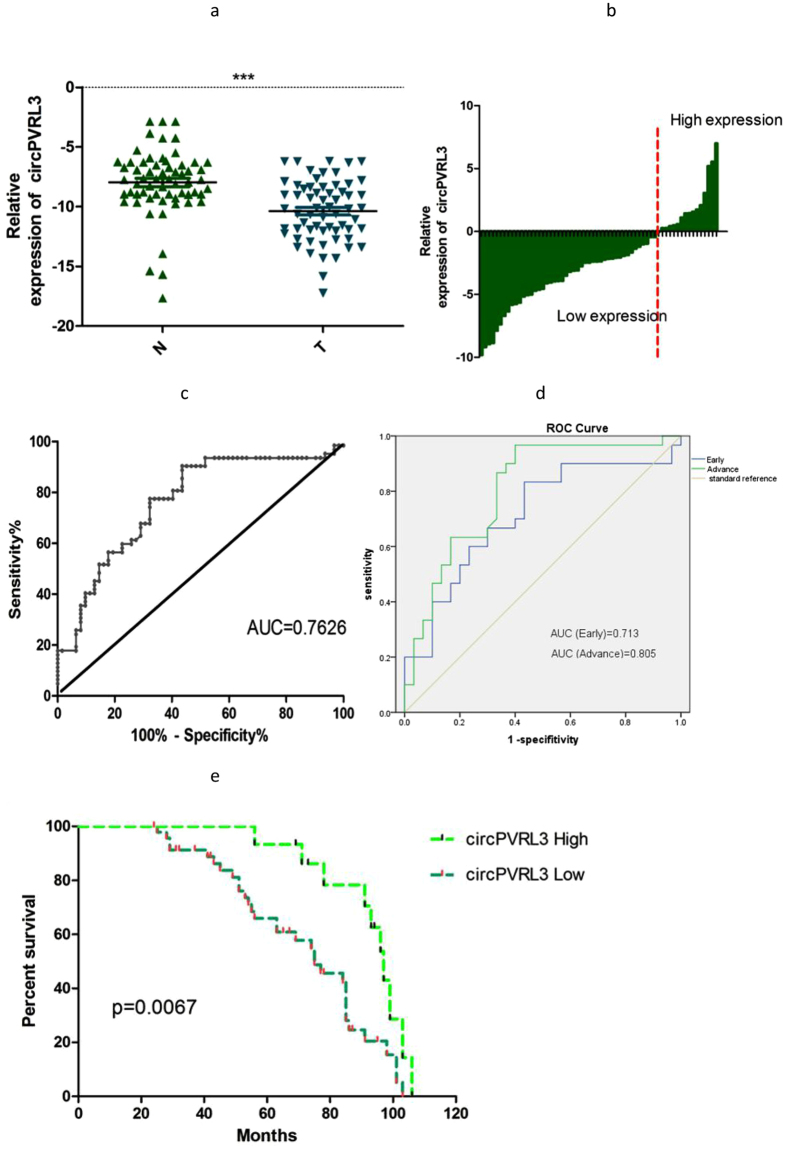
Relative circPVRL3 expression and its clinical significance in GC tissues. (**a**) The expression of circPVRL3 expression levels were significantly lower than that of adjacent noncancerous tissues from GC patients. The correlation of their −ΔCt value was determined. (**b**) The expression levels were divided into high-expression and low-expression. The expression of circPVRL3 in GC tissues higher than that of adjacent noncancerous tissues was defined as high-expression. In converse,the other group was defined as low-expression. The correlation of their −ΔCt value was determined. (**c**) The ROC curve has been used to evaluate circPVRL3 potential diagnostic value, the area under the ROC curve (AUC) was 0.7626. (**d**) The AUC of circPVRL3 expression in advanced (III-IV) TNM stages of GC was 0.805, higher than that in early (I-II) TNM stages. (**e**) Kaplan-Meier overall survival curve according to the circPVRL3 levels. Patients with low expression showed reduced survival time. ***P < 0.0001.

Using qRT-PCR, the circPVRL3 expression levels were defined for 62 paired primary cancerous and adjacent noncancerous tissues from GC patients and the results showed the expression of circPVRL3 in GC tissues were significantly lower than that of adjacent noncancerous tissues (Fig. 3a, P < 0.0001). Moreover, the levels were divided into high-expression and low-expression according to the difference between circPVRL3 expression in cancerous and adjacent noncancerous tissues. A total of 47 of these cases exhibited decreased levels of circPVRL3 in tumors compared with those observed in adjacent nontumorous tissues (Fig. 3b). Furthermore, we used the ROC curve to investigate the diagnostic value of circPVRL3 in distinguishing GC tissues from adjacent nontumorous tissues. When the expression level of circPVRL3 was analyzed for this purpose, the area under the ROC curve (AUC) was 0.7626 (Fig. 3c), with a sensitivity of 90.3% and specificity of 56.4%. We next focused on circPVRL3 association with TNM stage and prognostic and then performed ROC curve between the different TNM stages. Interestingly, the results showed the AUC of circPVRL3 expression in advanced (III-IV) TNM stages of GC was 0.805, higher than that in early (I-II) TNM stages (Fig. 3d).

In terms of the down-regulation of circPVRL3 in 62 GC patients, its association with clinicopathological features in GC patients was analyzed. As shown in Table 1, circPVRL3 level was not associated with age, gender, differentiation or lymphatic metastasis in patients with GC. However, down-expression of circPVRL3 level was negatively associated with TNM stage (Table 1, P < 0.05). Kaplan–Meier survival analysis and log-rank tests using patient postoperative survival were performed to further evaluate the correlation between circPVRL3 expression and prognosis of patients with GC. The results revealed that patients with lower levels of circPVRL3 expression had significantly shorter survival times than those with higher levels of circPVRL3 expression (Fig. 3e). Furthermore, univariate and multivariate analysis indicated that relative circPVRL3 expression level and TNM stage were each determined to be independent prognostic indicators for the overall survival rate of patients with GC (Table 2). These results revealed circPVRL3 might play a critical role in GC progression and development and could be applied as an independent biomarker for evaluating prognosis.

**Table 1:** Clinicopathological characteristics and expression of circPVRL3.

Variable	Case	Low expression	High expression	P-value
Age (year)				
≥60	52	39	13	0.545
<60	10	8	2	
Gender				0.553
Female	26	20	6	
Male	36	27	9	
Diameter				0.588
≥3(cm)	46	35	11	
<3(cm)	16	12	4	
Differentiation				0.189
low/middle	52	41	11	
well	10	6	4	
TNM Stage				0.026*
I-II	30	19	11	
III-IV	32	28	4	
Lymphatic metastasis				0.120
Yes	35	29	6	
No	27	18	9	
AFP				0.222
High	15	13	2	
Normal	47	34	13	
CEA				0.351
High	17	14	3	
Normal	45	33	12	
CA199				0.455
High	10	7	3	
Normal	52	40	12	
CA724				0.603
High	12	9	3	
Normal	50	38	12	

*P < 0.05, low- versus high-expression.

**Table 2:** Univariate and multivariate analysis for overall survival.

Variable	HR	95% CI	P-value
Univariate			
TNM stage	2.794	1.484–5.260	0.001*
circPVRL3 expression	0.356	0.168–0.755	0.007*
Multivariate			
TNM stage	2.311	1.209–4.418	0.011*
circPVRL3 expression	0.443	0.204–0.961	0.039*

*P < 0.05.

### Expression levels of circPVRL3 in GC cells and GC cell transfection

**Figure 4:**
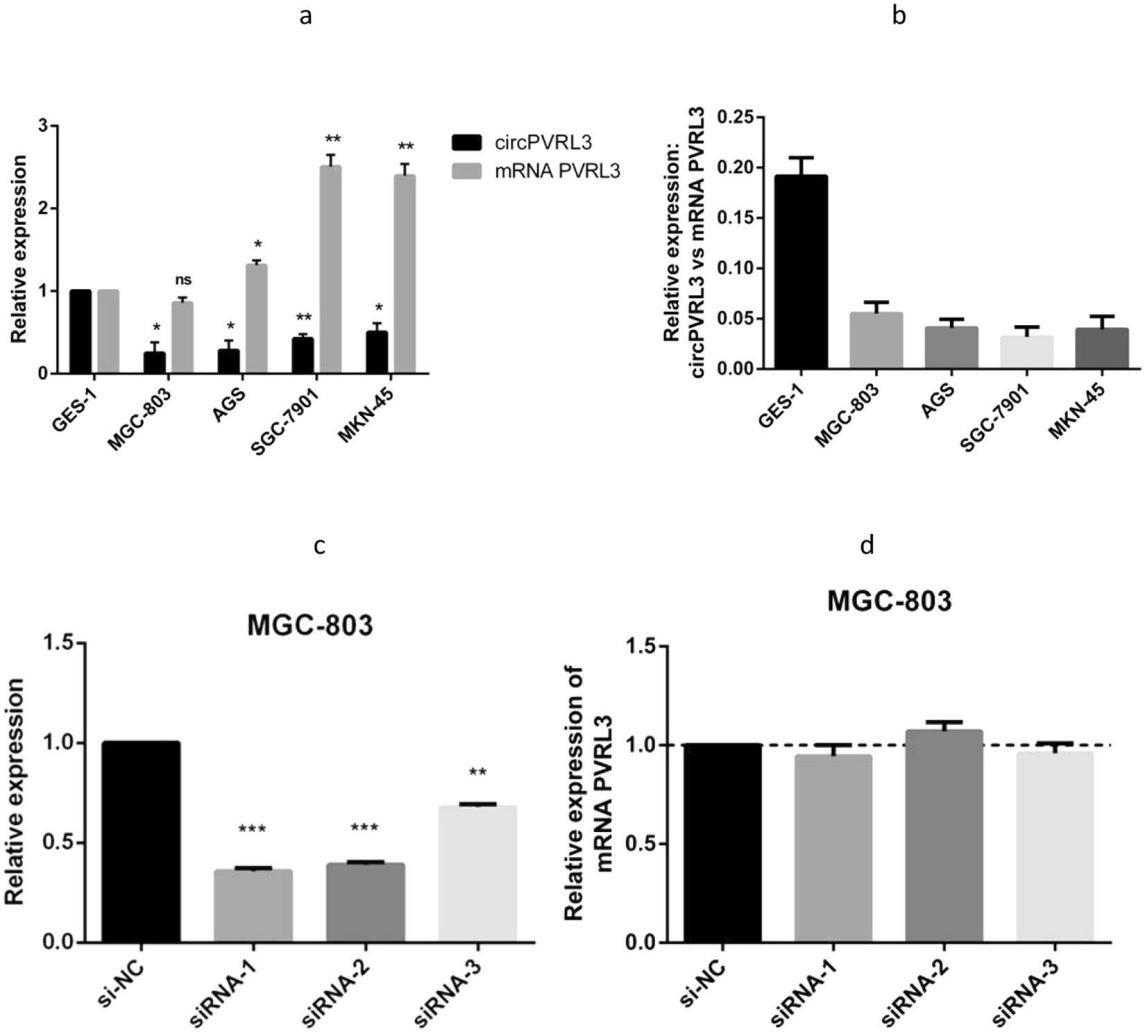
The expression of circPVRL3 in GC cell lines. (**a**) The expression of circPVRL3 in gastric cells was significantly lower than that in GES-1, the normal gastric epithelia cell line. The expression level of *PVRL3* mRNA in GC cell lines was higher than that in GES-1 except for MGC-803. (**b**) The abundance of circPVRL3 relative to *PVRL3* mRNA was significantly lower than that in GES-1. (**c**) Knock-down of circPVRL3 was confirmed via qRT-PCR, demonstrating the effective knockdown in MGC-803 cells. (**d**) *PVRL3* mRNA did not show significant difference when knockingdown of circPVRL3. *P < 0.05, **P < 0.01.

The expression of circPVRL3 and its parental mRNA in 4 GC cell lines was examined by qRT-PCR, revealing that the MGC-803 and AGS cells expressed the lowest levels of circPVRL3 expression compared with GES-1, the normal gastric epithelia cell line (Fig. 4a). In addition, the results showed that the expression level of *PVRL3* mRNA in GC cell lines was higher than that in GES-1 except for MGC-803 (Fig. 4a) and the abundance of circPVRL3 relative to *PVRL3* mRNA was determined, consistent with circPVRL3 expression results in GC cell lines (Fig. 4b). Next, MGC-803 cells were transfected with si-circPVRL3 or si-NC using the Lipofectamine 2000 transfection reagent. At 48 h after treatment, circPVRL3 expression was effectively knocked down in MGC-803 cells (Fig. 4c), wherase *PVRL3* mRNA did not show significant difference (Fig. 4d), providing evidence for ruling out the possibility that circPVRL3 siRNA could also affect *PVRL3* gene expression.

### Altered proliferation of GC cells by circPVRL3 expression level

**Figure 5:**
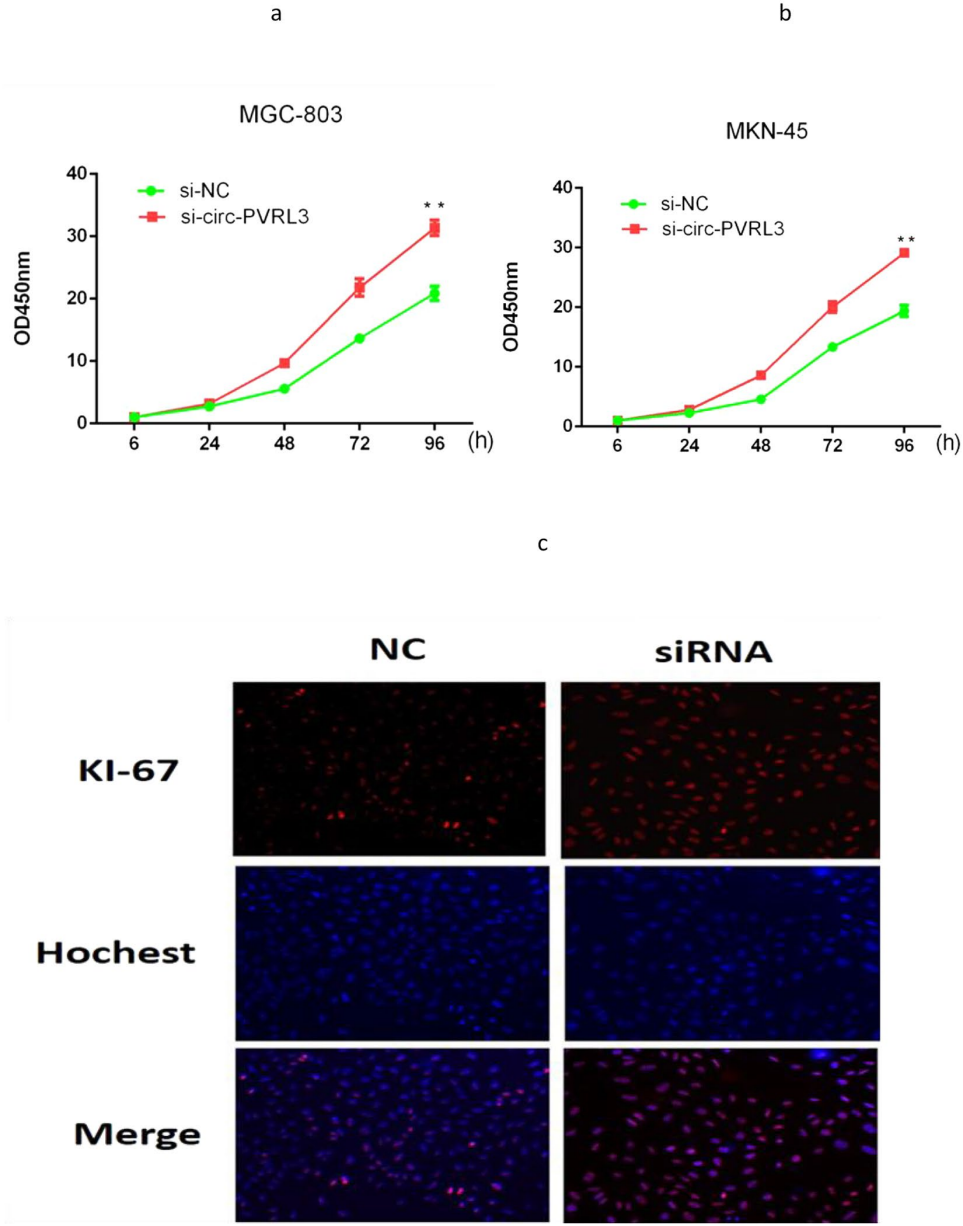
The expression of circPVRL3 in GC cell lines after knockdown. (**a**) Knockdown of circPVRL3 promoted MGC-803 cell proliferation significantly. (**b**) Knockdown of circPVRL3 promoted MKN-45 cell proliferation significantly. (**c**) Knockdown of circPVRL3 expression tended to have higher expression of Ki-67. **P < 0.01.

CCK8 assay was performed to detect the proliferation ability of MKN-45 and MGC-803 cells. Knockdown of circPVRL3 promoted cell proliferation significantly (Fig. 5a,b). Ki-67 is the most common biomarker of tumor proliferation, thus we performed immunofluorescence staining to further confirm that knockdown of circPVRL3 expression tended to have more positive expression of Ki-67 (Fig. 5c).

### Altered migration of GC cells by circPVRL3 expression level

**Figure 6:**
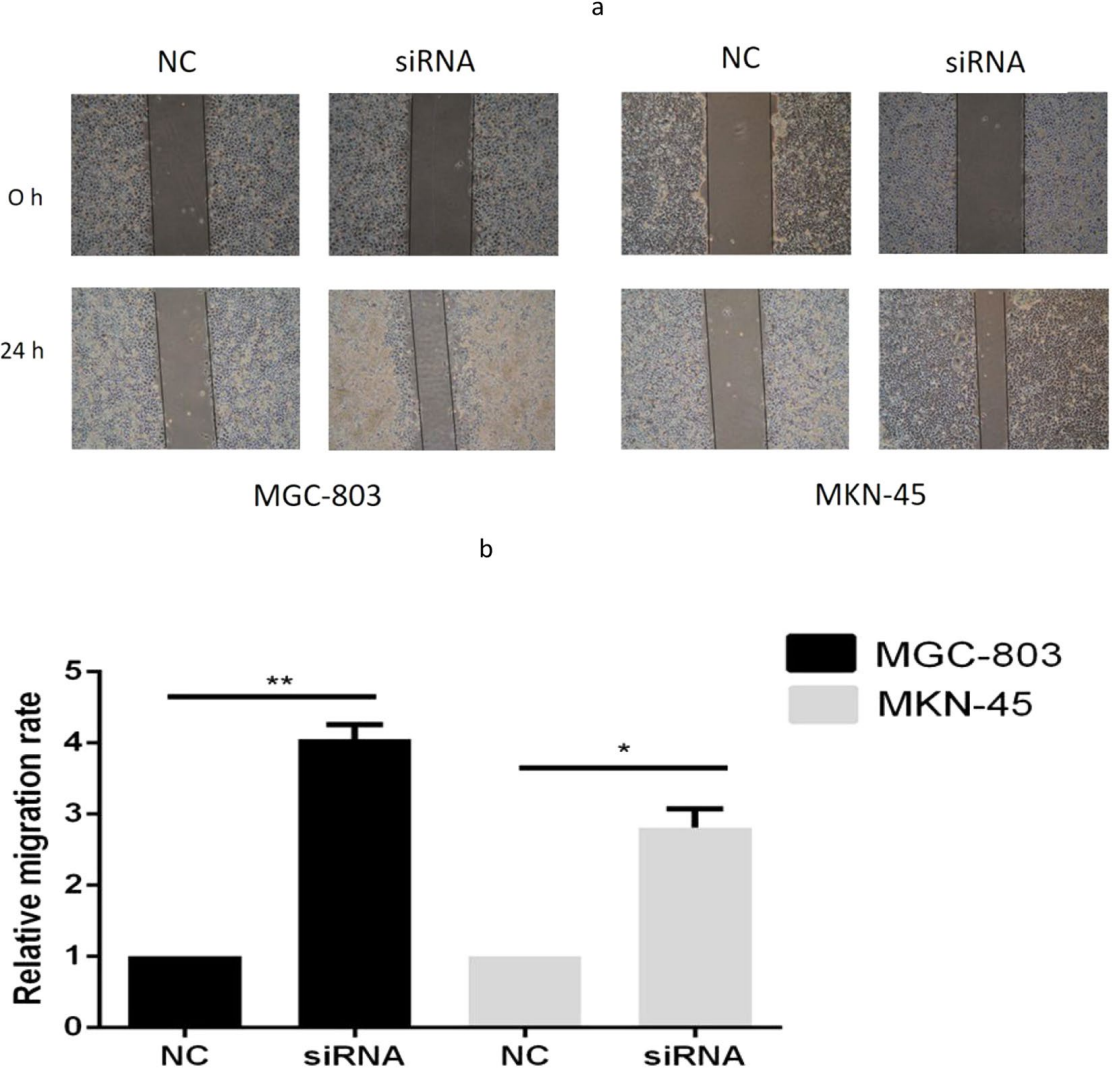
Scratch wound assay. (**a**) Inhibition of circPVRL3 by si-circPVRL3 produced a higher scratch closure rate than that treated with si-NC. (**b**) MGC-803 and MKN-45 showed a significantly higher relative migration rate after treatment with si-NC. The migration rate was calculated as the ratio of 0 h scratch distance to 24 h scratch distance. *P < 0.05, **P < 0.01.

The results of a scratch-wound assay in the confluent monolayer of the cultured GC cell line MKN-45 and MGC-803 demonstrated that suppression of circPVRL3 by si-circPVRL3 exhibited a higher scratch closure rate (Fig. 6a) and higher relative migration rate (Fig. 6b) compared with control cells treated with si-NC, respectively. These findings indicated that circPVRL3 may be closely associated with migration of GC cell lines.

### CircPVRL3 was abundant predominantly in the cytoplasm

**Figure 7:**
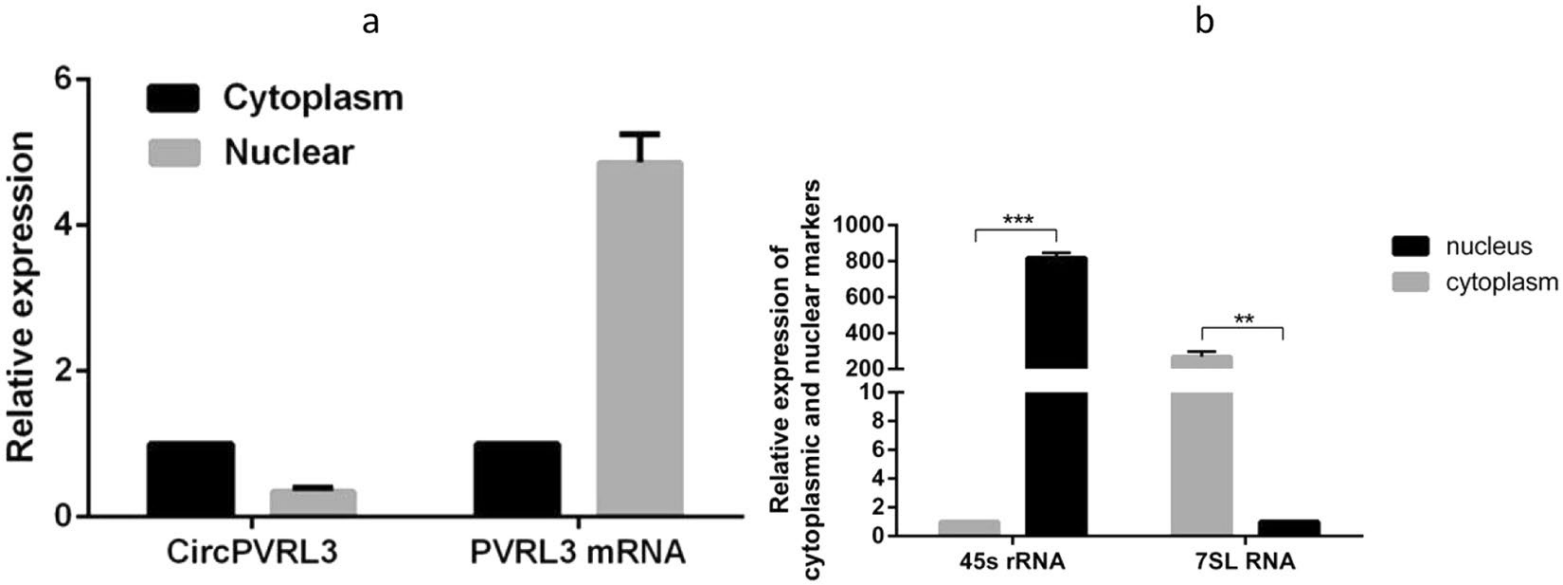
circPVRL3 RNA and fluorescence in nuclear cytoplasmic separation experiment demonstrated that the circular form of PVRL3 is preferentially localized in the cytoplasm.

The nuclear and cytoplasmic separation experiment demonstrated that the circPVRL3 was mainly localized in the cytoplasm but not in the nucleus (Fig. 7). This provides the most basic premise for circPVRL3 follow-up mechanism research.

### CircPVRL3 could serve as a sponge for miRNAs

**Figure 8:**
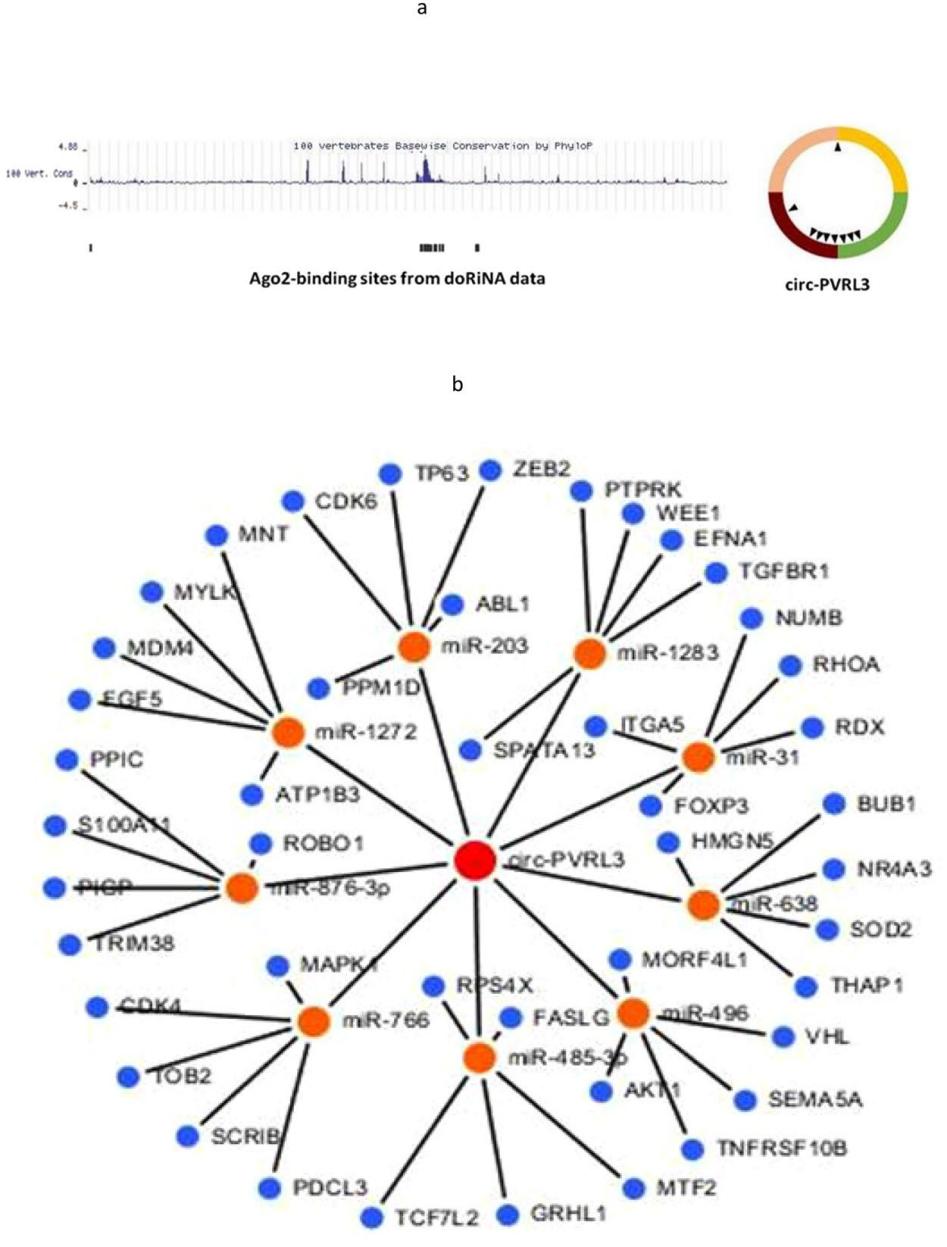
circPVRL3 serves as a sponge for multiple miRNAs. (**a**) AGO2 followed by high-throughput sequencing data from doRiNA revealed a high degree of AGO2 occupancy in the region of circPVRL3. (**b**) A schematic drawing showed the miRNAs associated with circPVRL3.

To investigate the possibility of circPVRL3 to bind to miRNAs, available sequencing data from doRiNA showed circPVRL3 has a high degree of AGO2 occupancy (Fig. 8a). To identify which miRNAs can bind to circPVRL3, we performed a circRNA-miRNA interaction network prediction on circinteractome database. A total of 9 miRNAs (i.e., miR-203, miR-1272, miR-1283, miR-31, miR-638, miR-496, miR-485-3p, miR-766, and miR-876-3p) and corresponding target mRNAs were predicted to have an interaction with circPVRL3 in this study. Specific details of the molecular interactions between circPVRL3 and its targets are depicted in Fig. 8b. Our prediction showed that circPVRL3 could bind to AGO2, FUS, LIN28A, PTB, and EIF4A3, and the detailed binding sites were listed in Table 3. These results indicated that the unique structure of circRNAs may also play an important role in the assembly of RNA or RBP complexes.

**Table 3:** circPVRL3 binding proteins.

RBP	Binding Sites
AGO2	1
FUS	2
LIN28A	2
PTB	3
EIF4A3	6

### CircPVRL3 has potential ability to encode protein

**Figure 9:**
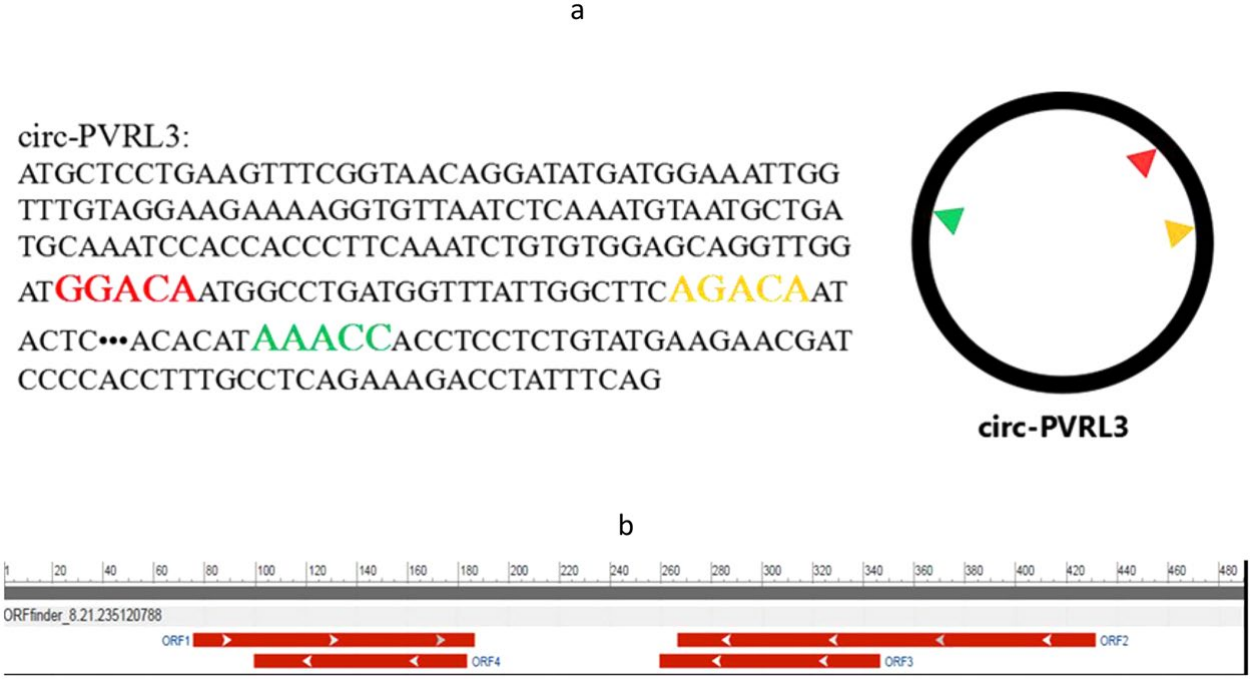
circPVRL3 has potential ability to encode protein. (**a**) circPVRL3 has the structure of m^6^A modification—RRm6ACH (R = G or A; H = A, C or U). (**b**) circPVRL3 has the structure of ORF.

CircPVRL3 contains an internal ribosome entry site (IRES), which can be efficiently translated and provides alternative, cap-independent translation initiation sites in eukaryotic cells (Table 4). Besides IRESs, open reading frame (ORF) is another key element to search newly sequenced circRNAs for potential protein-encoding segments. As shown in Fig. 9a, we made a prediction of circPVRL3 and fortunately found that circPVRL3 had an m^6^A modification structure with great translation potential. Our prediction results revealed that circPVRL3 has a structure of ORF (Fig. 9b). Based on the structure of IRES, ORF, and m^6^A modification, circPVRL3 may have the potential ability to encode proteins.

**Table 4:** circPVRL3 internal ribosomal entry sites.

Sequences producing significant alignments	Score (bits)	E Value
IRESite_Id:69	24	5.7
IRESite_Id:463	24	5.7
IRESite_Id:431	24	5.7
IRESite_Id:464	24	5.7
IRESite_Id:439	24	5.7
IRESite_Id:441	24	5.7
IRESite_Id:244	24	5.7
IRESite_Id:632	24	5.7
IRESite_Id:139	23	19
IRESite_Id:103	23	19

## Discussion

In the present study, we identified several circRNAs in gastric cancerous versus adjacent normal tissues, and characterized circPVRL3. This study was the first to indicate that circPVRL3 is stable but exhibits a decreased level in GC tissues compared with that in adjacent non-tumorous tissues with a sensitivity of 90.3% and specificity of 56.4%. However, the AUC of circPVRL3 represented a low capacity in distinguishing normal from cancerous tissue. Therefore we next focused on circPVRL3 association with TNM stage and prognostic and then performed ROC curve between the different TNM stages. Interestingly, the results showed the AUC of circPVRL3 expression in advanced stages of GC was higher than that in early stages, making it clearer that circPVLR3 could become a prognostic biomarker. Furthermore, down-expression of circPVRL3 was negatively associated with TNM stage and tended to have significantly shorter survival time compared with high-expression of circPVRL3. These results revealed that circPVRL3 may play a protection role in GC and could be applied as a powerful independent prognostic factor in future patient care.

Increasing evidence indicates that circRNA is not just a by-product of splicing errors, on the contrary, many circRNAs are thought to play important roles in the process of epithelial-mesenchymal transitions^19^. For example, the circular form of of CDR1 is able to sponge miR-135a in bladder cancer and exerts anti-carcinogenic functions^12^. Herein, we showed that inhibition of circPVRL3 expression promotes the proliferation and migration of GC cells. Nevertheless, we could not rule out the role of PVRL3 mRNA in the development of cancer, because it has also been reported to have a certain correlation with cancer^20^.

Competing endogenous RNA (ceRNA), including mRNAs, pseudogenic RNAs, long noncoding RNAs (lncRNAs) and circRNAs, are transcripts that cross-regulate each other by competing shared miRNAs. It has recently come to light that these circRNAs play critical roles in regulating gene expression by sequestering the miRNAs^21^. For example, Zhong and his colleges predicted that circTCF25 could sequester miR-103a-3p/miR-107 and demonstrated that over-expression of circTCF25 could down-regulate miR-103a-3p and miR-107, increase CDK6 expression, and promote proliferation and migration *in vitro* and *in vivo*^22^. Given that circPVRL3 is abundant in the cytoplasm, therefore, we supposed that the carcinogenic mechanisms of circPVRL3 may occur through their miRNA-mediated effects on gene expression in GC as well. A total of 9 miRNAs and corresponding target mRNAs were predicted to have an interaction with circPVRL3 in this study. Previous study has reported that the expression of miR-203 was significantly lower in gastric cancer samples compared to non-cancerous samples, closely correlated with advanced stage and lymph node involvement^23^ and could suppresses invasion of gastric cancer cells by targeting ERK1/2/Slug/E-cadherin signaling^24^. Zhang J and his colleagues reported microRNA-638 inhibits cell proliferation by targeting phospholipase D1 in human gastric carcinoma^25^. In addition, evidence showed that microRNA-31 could function as a suppressor regulated by epigenetic mechanisms in GC4^26^ and might target integrin α5 suppressing tumor cell invasion and metastasis by indirectly regulating PI3K/AKT pathway in human gastric cancer SGC7901 cells^27^. As for miR-1272, miR-1283,miR-496, miR-485-3p,miR-766 and miR-876-3p, no literature related to gastric cancer has been found in the current literature search.

It has been also shown that RNA-binding proteins (RBPs), such as argonaute and RNA polymerase II, can bind to circRNAs^7,28–30^. Some circRNAs can store, sort, or localize RBPs, and probably regulate the function of RBPs by acting as competing elements, in the same way as they modulate miRNA activity^31^. Therefore, we predicted that circPVRL3 is able to bind to AGO2, FUS, LIN28A, PTB, and EIF4A3, and may play an important role in the assembly of RNA or RBP complexes. Therefore, circPVRL3 might be used to bind and store components, to sort and deliver factors to particular subcellular locations or as scaffolds for the assembly of other complexes or reactions.

An intriguing possibility is that circRNAs could be translated to produce proteins, because most of circRNAs originate from exons and are localized in the cytoplasm^10^. N6 -methyladenosine (m^6^A) is the most abundant internal modification of RNAs in eukaryotes^32^. The modification preferably occurs in the consensus motif “RRm6 ACH” (R = G or A; H = A, C or U) and previous studies have reported m6A-driven translation of circRNAs is widespread, with hundreds of endogenous circRNAs having translation potential^33^. If the conserved sequence is present in circRNAs, it is possible to initiate the protein translation process through this mechanism. In addition, inclusion of IRES and ORF allows translation of engineered circRNAs, some circRNAs could be translated^34^. In this study, we performed a prediction and found that circPVRL3 has a structure of IRES, ORF, and m^6^A modification, indicating that circPVRL3 may have the potential ability to encode proteins. It is interesting to know the nature of possible proteins encoded by circRNAs. If such translational products would exist endogenously, they may exert specific biological effects or interfere with protein-protein interactions. However, it remains unclear whether and how these circRNAs are translated into protein under normal conditions. As more functions of circRNAs are discovered, the widespread use of this newly circRNA named circPVRL3 would emerge in the future.

Several limitations should not be ignored when interpreting the results. Firstly, all experimental samples are from one hospital, it is recommended that there are more hospital samples or even different races of the samples being studied. Secondly, identification of the relative molecules, such as interactions between circRNAs and miRNAs, are suggested for further experiment confirmation. As a result, further biologic and functional evidence is needed to confirm the effects of circPVRL3 on gastric cancer.

In summary, our findings suggest that down-regulation of circPVRL3 could promote the proliferation in GC, exerting carcinogenesis roles by sponging miRNAs. More than that, circPVRL3 has the potential ability to encode proteins.

### Ethics approval and consent to participate

The local medical ethics committee approved the study protocol.

### Consent for publication

All authors agree to publish the manuscript.

## References

[1] Nagini S (2012). Carcinoma of the stomach: A review of epidemiology, pathogenesis, molecular genetics and chemoprevention. World J Gastrointest Oncol..

[2] Chen W (2016). Cancer statistics in China, 2015. CA Cancer J Clin..

[3] Yagi K (2012). Diagnosis of Early Gastric Cancer by Magnifying Endoscopy with NBI from Viewpoint of Histological Imaging: Mucosal Patterning in terms of White Zone Visibility and Its Relationship to Histology. Diagn Ther Endosc..

[4] Narikazu B (2011). Past and Present Achievements, and Future Direction of the Gastrointestinal Oncology Study Group (GIOSG), a Division of Japan Clinical Oncology Group (JCOG). Jpn J Clin Oncol..

[5] Terracciano D (2017). The role of a new class of long noncoding RNAs transcribed from ultraconserved regions in cancer. Biochim Biophys Acta..

[6] Suzuki H, Tsukahara T (2014). A view of pre-mRNA splicing from RNase R resistant RNAs. Int. J. Mol. Sci..

[7] Memczak S (2013). Circular RNAs are a large class of animal RNAs with regulatory potency. Nature..

[8] Salzman J (2013). Cell-type specific features of circular RNA expression. PLoS Genet..

[9] Li Z (2015). Exon-intron circular RNAs regulate transcription in the nucleus. Nat. Struct. Mol. Biol..

[10] Hentze MW, Preiss T (2013). Circular RNAs: splicing’s enigma variations. EMBO J..

[11] Wang Y, Wang Z (2015). Efficient backsplicing produces translatable circular mRNAs. RNA..

[12] Li Y (2015). Circular RNA is enriched and stable in exosomes: a promising biomarker for cancer diagnosis. Cell Res..

[13] Barrett SP, Salzman J (2016). Circular RNAs: analysis, expression and potential functions. Development..

[14] Wang K (2017). Androgen receptor (AR) promotes clear cell renal cell carcinoma (ccRCC) migration and invasion via altering the circHIAT1/miR-195-5p/29a-3p/29c-3p/CDC42 signals. Cancer Lett..

[15] Chen J (2017). Circular RNA profile identifies circPVT1 as a proliferative factor and prognostic marker in gastric cancer. Cancer lett..

[16] Li T (2018). Plasma circular RNA profiling of patients with gastric cancer and their droplet digital RT-PCR detection. J Mol Med..

[17] Li P (2015). Using circular RNA as a novel type of biomarker in the screening of gastric cancer. Clin Chim Acta..

[18] Feng Y (2017). Naturally existing isoforms of miR-222 have distinct functions Naturally existing isoforms of miR-222 have distinct functions. Nucleic Acids Research..

[19] Neumann DP, Goodall GJ, Gregory PA (2017). Regulation of splicing and circularisation of RNA in epithelial mesenchymal plasticity. Semin Cell Dev Biol..

[20] Zhou L (2010). Silencing of thrombospondin-1 is critical for myc-induced metastatic phenotypes in medulloblastoma. Cancer Res..

[21] Rusinek D (2015). BRAFV600E-associated gene expression profile: Early changes in the transcriptome, based on a transgenic mouse model of papillary thyroid carcinoma. PLoS One..

[22] Gao D (2017). Screening circular RNA related to chemotherapeutic resistance in breast cancer. Epigenomics..

[23] Zhong Z, Lv M, Chen J (2016). Screening differential circular RNA expression profiles reveals the regulatory role of circTCF25-miR-103a-3p/miR-107-CDK6 pathway in bladder carcinoma. Sci Rep..

[24] Zheng Y (2017). The expression level of miR-203 in patients with gastric cancer and its clinical significance. Pathol Res Pract..

[25] Gao P (2017). microRNA-203 suppresses invasion of gastric cancer cells by targeting ERK1/2/Slug/E-cadherin signaling. Cancer Biomark..

[26] Zhang J (2015). MicroRNA-638 inhibits cell proliferation by targeting phospholipase D1 in human gastric carcinoma. Protein Cell..

[27] Wei J (2017). MicroRNA-31 Function as a Suppressor Was Regulated by Epigenetic Mechanisms in Gastric Cancer. Biomed Res Int..

[28] Zhang X (2017). Upregulation of microRNA-31 targeting integrin α5 suppresses tumor cell invasion and metastasis by indirectly regulating PI3K/AKT pathway in human gastric cancer SGC7901 cells. Tumour Biol..

[29] Hansen TB (2013). Natural RNA circles function as efficient microRNA sponges. Nature..

[30] Zhang Y (2013). Circular intronic long noncoding RNAs. Mol Cell..

[31] Ashwal-Fluss R (2014). circRNA biogenesis competes with pre-mRNA splicing. Mol Cell..

[32] Pamudurti NR (2017). Translation of circRNAs. Mol Cell..

[33] Zhou C (2017). Genome-Wide Maps of m6A circRNAs Identify Widespread and Cell-Type-Specific Methylation Patterns that Are Distinct from mRNAs. Cell reports..

[34] Yang Y (2017). Extensive translation of circular RNAs driven by N6-methyladenosine. Cell Res..

